# A FAIRNESS LENS TO LATER-LIFE PLANNING

**DOI:** 10.1093/geroni/igz038.1591

**Published:** 2019-11-08

**Authors:** Marlene Stum

**Affiliations:** University of Minnesota, St. Paul, Minnesota, United States

## Abstract

Being “fair” is often a desired goal when individuals are planning for financing future care, leaving an inheritance, and selecting an attorney-in-fact for financial and/or health care. However, differing perceptions about the fair use of an older parent’s resources (paying for formal care, leaving an inheritance, compensating family caregivers, rewarding a sense of entitlement), and issues of who can or should be involved in decision processes, can lead to avoiding planning and be a source of family conflict. The complexities of fair decision rules for distributing resources, and important criteria for determining fair decision processes will be discussed, guided by interpersonal social justice theories, and findings from a qualitative study of inheritance involving older adults and adult children from the same family system (N= 18). Implications for helping family members, and professionals working with them, navigate “being fair” and increase later life planning will be shared.

